# Mapping microhabitat thermal patterns in artificial breakwaters: Alteration of intertidal biodiversity by higher rock temperature

**DOI:** 10.1002/ece3.5776

**Published:** 2019-11-04

**Authors:** Moisés A. Aguilera, René M. Arias, Tatiana Manzur

**Affiliations:** ^1^ Departamento de Biología Marina Facultad de Ciencias del Mar Universidad Católica del Norte Coquimbo Chile

**Keywords:** artificial breakwaters, boulder fields, coastal microclimate, rocky intertidal, thermal patterns, urbanization

## Abstract

Urbanization is altering community structure and functioning in marine ecosystems, but knowledge about the mechanisms driving loss of species diversity is still limited. Here, we examine rock thermal patterns in artificial breakwaters and test whether they have higher and spatially less variable rock temperature than natural adjacent habitats, which corresponds with lower biodiversity patterns. We estimated rock temperatures at mid‐high intertidal using infrared thermography during mid‐day in summer, in both artificial (Rip‐raps) and natural (boulder fields) habitats. We also conducted diurnal thermal surveys (every 4 hr) in four seasons at one study site. Concurrent sampling of air and seawater temperature, wind velocity, and topographic structure of habitats were considered to explore their influence on rock temperature. Rock temperature was in average 3.7°C higher in the artificial breakwater in two of the three study sites, while air temperature was about 1.5–4°C higher at this habitat at summer. Thermal patterns were more homogeneous across the artificial habitat. Lower species abundance and richness in the artificial breakwaters were associated with higher rock temperature. Mechanism underlying enhanced substrate temperature in the artificial structures seems related to their lower small‐scale spatial heterogeneity. Our study thus highlighted that higher rock temperature in artificial breakwaters can contribute to loss of biodiversity and that integrated artificial structures may alter coastal urban microclimates, a matter that should be considered in the spatial planning of urban coastal ecosystems.

## Graphical abstract

We evaluated substrate thermal patterns in artificial breakwaters and natural adjacent boulder fields. We found higher rock and air temperature in the artificial habitat. Species abundance and richness were lower in the artificial habitat and related negatively with rock temperature.

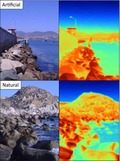

## 1 INTRODUCTION

Urbanization is altering community structure and functioning in both terrestrial and marine ecosystems worldwide (Alberti et al., 2017; Bulleri & Chapman, 2010; Firth et al., 2016; Forman, 2014; Gaston, 2013). Particularly, urban infrastructure is often associated with enhanced thermal patterns, which can affect human health, and plant and animal distribution and abundance in terrestrial ecosystems (Larsen, 2015; Wilby & Perry, 2006). Thus, mapping the thermal mosaics found within urban environments such as cities and city boundaries can help to develop specific mitigation strategies to cope with the urban “heat island” effects and its impact on local biodiversity (Kuttler, 2008; Larsen, 2015; Oke, 1989). Few attempts, however, have been made to determine the variation of temperature associated with urban coastal marine structures and their associated impacts on marine coastal community structure.

In marine coastal ecosystems, air, substrate, and seawater temperature play a major role as main drivers of both benthic and pelagic species' distribution, abundance, and life‐history traits at local and global scales (e.g., Helmuth et al., 2006; Lima et al., 2016; Petes, Mouchka, Milston‐Clements, Momoda, & Menge, 2008; Sunday, Bates, & Dulvy, 2012). Variation in thermal patterns can have significant impacts on the strength of biotic interactions and concomitantly on community structure (Broitman, Mieszkowska, Helmuth, & Blanchette, 2008; Broitman, Szathmary, Mislan, Blanchette, & Helmuth, 2009; Kordas, Harley, & O'Connor, 2011; Monaco, Wethey, & Helmuth, 2016). Spatial variation in heat and desiccation stress, for example, has been shown to affect both mobile and sessile species distributions (Helmuth et al., 2006, 2002; Lima et al., 2016; Seabra et al., 2011). Given the increasing frequency and effects of environmental fluctuations like “heat waves” in temperate systems (Wernberg et al., 2012), many species have been strongly impacted by high levels of air desiccation stress, or increase in seawater temperature, often causing massive mortalities and community phase shifts (Garrabou et al., 2009; Wernberg et al., 2016, 2012). There is, therefore, growing concern about how changes in temperature regimes are modified by local anthropogenic factors, which will subsequently affect patterns of species distribution and composition.

The addition of built coastal infrastructures is causing a transformation of coastal landscapes with an associated decline of biodiversity, homogenization of biota and increase in the frequency of nonindigenous, exotic species (e.g., Airoldi, Turon, Perkol‐Finkel, & Rius, 2015; Bulleri & Chapman, 2010; Dafforn et al., 2015; Firth et al., 2016). Construction of structures such as concrete seawalls, granite breakwaters, groynes, jetties, and boat ramps, for example, are increasing in most countries for multiple purposes (Aguilera, 2018; Bulleri & Chapman, 2010; Firth et al., 2014; Gittman et al., 2015; Moschella et al., 2005; Waltham & Sheaves, 2015). These structures can differentially affect coastal ecosystems, but the general pattern observed is that they harbor fewer species compared with natural (reference) habitats (Firth et al., 2016, 2014; Perkins, Ng, Dudgeon, Bonebrake, & Leung, 2015) and alter species composition by reducing settlement of habitat‐forming species (Aguilera, Broitman, & Thiel, 2014; Firth et al., 2014), disrupting species interactions (Ferrario, Iveša, Jaklin, Perkol‐Finkel, & Airoldi, 2016; Iveša, Chapman, Underwood, & Murphy, 2010; Klein, Underwood, & Chapman, 2011), enhancing presence of invasive species (Airoldi et al., 2015; Bulleri & Airoldi, 2005; Bulleri, Airoldi, Branca, & Abbiati, 2006), and affecting connectivity (Bishop et al., 2017) and the functional structure of marine ecosystems (Mayer‐Pinto et al., 2018). In part, loss of species richness on these infrastructures seems related to their low spatial heterogeneity as compared with natural shores (Aguilera et al., 2014; Coombes, La Marca, Naylor, & Thompson, 2015; Firth et al., 2014, 2013). Specifically, the reduction in frequency of topographic elements related to spatial heterogeneity, like rocks or tide pools, crevices, pits and/or depressions, in artificial infrastructures have been considered one of the main factors related to the reduction in abundance of “rare” and habitat‐forming species in these habitats (Coombes et al., 2015; Liversage, Cole, Coleman, & McQuaid, 2017; Loke, Bouma, & Todd, 2017; Loke, Ladle, Bouma, & Todd, 2015; Martins, Jenkins, Neto, Hawkins, & Thompson, 2016). These topographic features are microhabitats, which function as refuges to reduce thermal stress for both invertebrate and algal species (Garrity, 1984; Williams & Morritt, 1995) although rock pools can also become stressful environments in terms of temperature and salinity (Chan, 2000; Firth & Williams, 2009; Morritt et al., 2007). Thus, strategies to enhance the ecological value of artificial infrastructures in different latitudes are being made based on the provision of these refuges (Strain et al., 2018).

The spatial homogeneity which characterizes artificial infrastructures can, therefore, impose a great challenge for intertidal species that live in these habitats (Chapman, 2003, 2006, 2012), which have developed a suite of behavioral strategies like aggregation, that is, either inside or outside crevices (Aguilera & Navarrete, 2011; Cartwright & Williams, 2012; Chapperon & Seuront, 2011a, 2011b; Garrity, 1984; Harper & Williams, 2001; Moreira, Chapman, & Underwood, 2007), aggregating towering and mushrooming (Ng et al., 2017; Williams et al., 2005), sun orientation shell movement (Muñoz, Randall Finke, Camus, & Bozinovic, 2005), adopt sloping or vertical shaded habitats (Lima et al., 2016; Miller, Harley, & Denny, 2009), or take refuge underneath boulders (Liversage, 2016) to cope with wave and thermal stress. This lack of suitable habitat heterogeneity seems especially relevant in artificial breakwaters made of granite boulders or “rip‐raps” which, despite their reduced small‐scale (few cms) spatial heterogeneity, have increased structural complexity at larger scales (tens of meters). This large‐scale complexity is produced by the presence of irregular interstices or gaps left between “rip‐rap” joints, which can be used as refuge by medium to large size species like crabs or rats (Aguilera, 2018). Presently, there is scarce information about the thermal patterns of these artificial structures (Seuront et al., 2018), and how these influence the abundance, distribution, and occurrences of intertidal species. Higher and more homogeneous substrate temperature in artificial breakwaters may have a primary role on supporting less species compared with natural habitats.

Here, we examine substrate temperature patterns on artificial breakwaters made of granite boulders to evaluate if the spatial variation in thermal patterns could correlate with patterns of intertidal species abundance, distribution and richness. In addition, we examined the diurnal variability of thermal patterns in both artificial and natural habitats such as boulder fields, which are analogue of their whole structural complexity, to evaluate whether the artificial habitat has the potential to emit more heat at evening and night and thus having higher rocky and air temperature than natural adjacent habitats. We hypothesized that 1) mean substrate temperature should be higher, but spatially less variable, in artificial breakwaters than in natural habitats (e.g., boulder fields) given their lack of small‐scale (few cm) spatial heterogeneity and that 2) thermally homogeneous patterns on the artificial infrastructure would be correlated to lower species abundance and richness in this habitat compared with the natural habitats.

In Chile, more than 45% of the human population lives in coastal cities with higher densities concentrated between 29° and 35°S (Hidalgo, Arenas, & Monsalve, 2009). Coastal armoring is one of the most important but less recognized management conflicts in present‐day Chile and is proceeding either to expand human settlements as disaster prevention/mitigation infrastructure, or for the development of new activities related to mining, tourism, deep‐water harbors or leisure activities (Aguilera, 2018). To date, very few attempts have been made to incorporate coastal planning of artificial infrastructure management into the legal framework of the country, like in most South American countries (de Andrés, Barragán, & Scherer, 2018; Barragán & de Andrés, 2016).

## 2 METHODS

### 2.1 Study system

**Figure 1:**
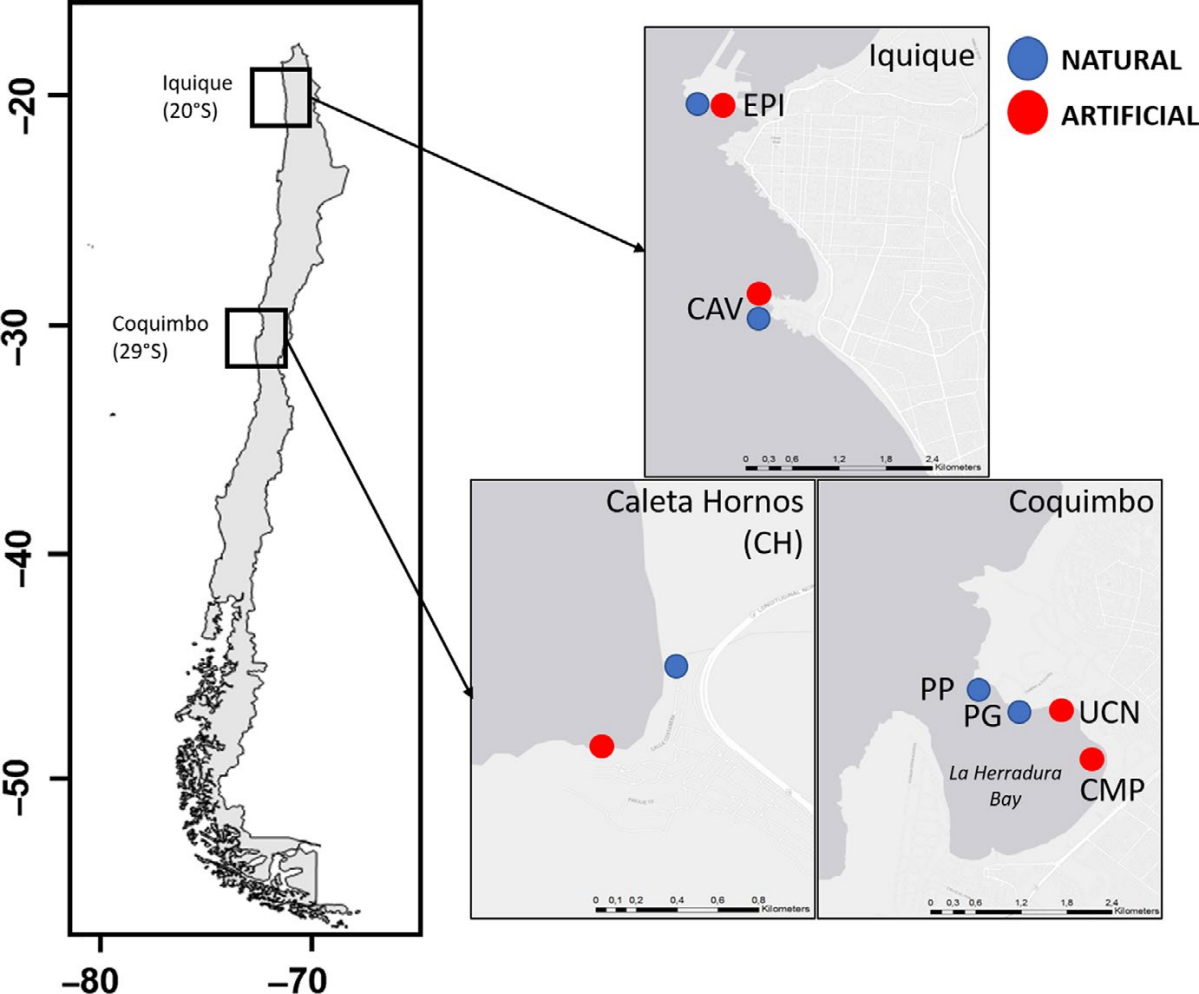
Study sites. Location of the different study sites and habitats in Coquimbo (29°S) and Iquique (20°S), and small town (Caleta Hornos, CH) located close to Coquimbo city (~40 km from the La Herradura Bay). Right panel show the specific location of artificial breakwaters (red) and natural habitats (blue) considered in the study. Sites located in Iquique (20°S) were only considered for the substrate thermal pattern surveys

We considered two study sites in Coquimbo region (29°S), La Herradura (29°58′S‐71°20′W), located in Coquimbo city and Caleta Hornos (29°62′S‐71°29′W), about 50 km north from La Herradura (see Figure 1). Natural boulder fields are also common in these sites and resemble (~1 m^2^ boulder size) the artificial breakwaters but differ from them in shape (i.e., they are more ovoid than rip‐raps). Along this coast, ports and harbors are associated with large (100–300 m long) artificial breakwaters made of granite boulders (1 m^2^ rip‐raps). Rip‐raps present in the study sites were built mostly with diorite and gabbro rocks, quarried from the coast near Coquimbo city. Thus, they resemble in geochemical composition to natural boulders found in the study locality (e.g., have biotite and hornblende minerals). Thus, these artificial habitats seem relatively similar in large‐scale spatial structure and geochemical composition but are different in topographic features like rock shape and surface topography (e.g., differences in pits and crevices availability) compared to the natural boulder fields present at the study sites (see Figure S1 in Appendix). Differences in color between natural rocks and rip‐raps (brownish vs. clear, respectively) seem directly related to presence sessile species and microalgae cover and bare rock (see main Results).

The Coquimbo region, where most part of the study was conducted, experiences a more temperate four‐season weather system characterized by a frequent southerly wind and cloud accumulation compared with zones north of this latitude (Rahn, 2012; Rahn & Garreaud, 2011). During the afternoon, the coastal southerly winds reinforce which contrast with the alongshore warm air advection from heated land (Rahn & Garreaud, 2011). The port area of Coquimbo city is characterized by average air temperatures which fluctuates from 15.4°C in autumn, 12.5°C in winter, and 14.4°C in spring, with summer temperatures fluctuating from a maximum of 21.7°C to a minimum of 9°C (CEAZA‐met, 2018). Seawater temperatures fluctuate from 9 to 18°C (Valle‐Levinson et al., 2000).

We investigated the thermal patterns of both artificial breakwaters and natural boulder fields in the three study sites at Coquimbo (29°S), and additionally, we consider two sites at the locality of Iquique (20°S), which is a city port with a more subtropical climate (Figure 1). At these sites, rip‐raps and natural boulders are larger than in Coquimbo (~2.25 m^2^), but they are relatively similar in mineral concentration and color. This last locality was considered to test the generality of the artificial–natural differences in rocky temperature given this experiences much higher temperatures during summer than Coquimbo (i.e., averages fluctuates from 19 to 25°C). At Coquimbo, we considered three artificial breakwaters, one associated with a mining port (Compañía Minera del Pacífico; hereafter CMP), another located in the wharf of the Universidad Católica del Norte (hereafter UCN), both at La Herradura Bay, and another located in a fishermen wharf located in Caleta Hornos (CHa) (Figure 1). Correspondingly, three natural boulder fields close (less or 600 m away) to the artificial habitats were selected: Pérgola (PG) and La Pampilla (PP) at La Herradura Bay, and one site close to Caleta Hornos (CHn) (Figure 1). Thus, we considered paired “artificial–natural” habitat comparison at each locality, that is, CMP‐PG, UCN‐PP, and CH_a‐n_. At Iquique, we selected two artificial breakwaters and a corresponding natural adjacent rocky habitat at two sites: one located in the shipping port of the city (EPI_a‐n_) and another in the Cavancha Península (CAV_a‐n_) (both sites separated by about 3 km). All sites were selected based on accessibility and similarity in rip‐rap dimensions. The study was conducted in the mid‐high intertidal zone during low tide, thus reducing the potential effect of frequent rock moisture on thermal patterns.

### 2.2 Spatial‐temporal variation in thermal patterns

To characterize the thermal patterns of both artificial and natural habitats at the different study sites, we estimated the rock temperature at different locations of the rip‐raps and boulders sites. We selected four groups of two rip‐rap or boulders across each artificial breakwater and boulder fields (see Figure S2A in Appendix). In each group, we selected two zones on individual rip‐rap and boulders; either the “side” or “top,” and another zone located in the intersection (“union”) between two rip‐raps or boulders (Figure S2A). In zones “top” and “sides,” two 20 × 20 cm quadrats were randomly located, while in zone “union,” one quadrat was located given limitations of space between boulders and feasibility to correctly estimate thermal patterns there. In all quadrats, we estimated the substrate temperature of the area using thermal images (see below). In total, we estimated 325 rock temperatures throughout the study. Each natural–artificial habitat pair (see Figure 1) was sampled at the same day with about 30 min of differences between each survey. Boulders and rip‐raps sampled in the study were similar in orientation (most were northwest‐oriented, see Figure S3 in Appendix), and thus, wind exposure (southwest) was expected to be similar between the considered habitat types (Figure S3).

We used infrared thermal imaging as a noncontact and noninvasive technique for temperature measurement (Lathlean & Seuront, 2015; Lathlean, Seuront, & Ng, 2017; Seuront et al., 2018). This method is widely used in a range of fields, especially in intertidal rocky shore systems (Chapperon & Seuront, 2011a, 2011b; Lathlean, Ayre, & Minchinton, 2012; Lathlean & Seuront, 2015; Rojas et al., 2013), and allows measurement of the complex thermal patterns of natural and/or artificial surfaces at micro‐scales (few cm's) (Seuront et al., 2018). We used this technique because we were focused on the spatial patterns of substrate temperature instead of organisms' tissue temperatures, for which data loggers or thermocouples would be more appropriated (see Judge, Choi, & Helmuth, 2018). Notwithstanding, we use HOBO *TidbiT v2* temperature logger installed in each site to complement recording of temperature temporal variability. Thermal images were obtained with a Fluke Ti110 camera (Fluke Corporation, Everett, WA, USA). The thermal sensitivity of the thermal camera is <0.021C at 30°C, and the temperature measurement accuracy is 2%. Emissivity values of the substrata were set at 0.95 appropriated for rocky substrate and following previous studies (Chapperon & Seuront, 2012; Lathlean et al., 2017). Images were subsequently analyzed in the laboratory using Fluke Smart View software version 3.15 (Fluke Corporation, USA, 2016) (see Figure S4). In addition, to examine the variability of the diurnal thermal pattern (hereafter “diurnal surveys”), we selected one artificial breakwater and the corresponding boulder field located at La Herradura Bay at Coquimbo, and conducted the same protocol during different day phases: sunrise (8:00 hr), mid‐day (12:00 hr), afternoon (16:00 hr), and evening (20:00 hr). Each sampling conducted during the different day phases, at both habitat types, was conducted at the same day with a maximum of 10–12 min of differences between habitats for a corresponding day phase (it should be noted that sites were separated by less than 600 m). Diurnal surveys were conducted in two random days during summer (January), autumn (May), winter (August), and spring (November) of 2018. Autumn and winter sampling included partial cloudy days, while spring and summer included completely sunny days. Sampling was conducted at the same rip‐raps and boulder field groups at different seasons.

### 2.3 Environmental and topographic variables

Concurrently with thermal pictures and at each site and habitat, we estimated (1) air temperature and wind velocity measured between 0.5 and 1.5 m above the substrate surface with a digital thermometer and anemometer, respectively; (2) seawater temperature (1 m depth) with a digital thermometer (*Extech TM25*); (3) rock slope of each quadrat at each sampled rip‐rap or boulder with a digital inclinometer (Bosch Professional GIM60L*)*; (4) orientation (south–north, east–west) of each group of rip‐raps or boulders with an analogue compass, and (5) topographic complexity measured at two scales; (a) among boulder or rip‐rap which we called “structural complexity” (see Figure S2A in Appendix) and (b) at the within boulder scale (i.e., at each quadrat), using the “link‐chain” method (Beck, 1998).

### 2.4 Species abundance and richness

At each 20 × 20 cm quadrat positioned in the different boulder and rip‐rap zones at different time, we estimated the density and percent cover of invertebrates and algae, respectively, by means of digital pictures (Canon PowerShot 30 Mega Pixels). This study was conducted only for the locality of Coquimbo. Density of mobile species was estimated in situ and corroborated through digital photograph analyses. Percentage cover of algae and sessile invertebrates was analyzed using the software CPCe (Coral Point Count with Excel extensions) (Kohler & Gill, 2006).

### 2.5 Statistical analyses

To examine the effect of habitat type (artificial/natural) and position sampled to the mean substrate temperature (ST_mean), and to deal with differences in sample size between union versus the other positions (i.e., side and top) (Bates, Mächler, Bolker, & Walker, 2015; Bolker et al., 2009) we used a random‐intercept general linear mixed model (LMM) including habitat type (two levels: artificial, natural; fixed), site (random and nested in habitat type), and position (side, top, union; fixed and orthogonal to habitat type) as explanatory factors. Individual boulder groups sampled at each habitat was considered the random factor. The three sampling dates were pooled for analyses. We also constructed a “null” model (only with the random factor) in order to calculate the marginal and conditional pseudo‐*R* ^2^ as coefficients of determination of the main model, where the first represents the variability accounted for the fixed component of the model and the second represents the variability accounted for the entire model (i.e., both fixed and random effects included). These analyses were performed using the library *MASS, MuMIn,* and *lme4* in the R programming environment version 3.5.0 (Bates et al., 2015; R Core Team, 2018).

We expected that topographic elements related to thermal microhabitats (crevices, depressions, pits, etc.) had an effect (i.e., reduction or increase) especially on minimal substrate temperature recorded. Thus, we evaluated the effect of topographic complexity (small scale) and rock slope on minimum substrate temperature (ST_min) recorded on each position for both habitat type, with a multiple linear regression model using slope and structural complexity as explanatory variables. This model allowed us to explore the effects of the interaction between slope and complexity on ST_min considering both habitat types. We evaluate the effects of mean rock temperature on biotic variables like abundance and species richness, with a polynomial regression model (order = 2) and with a simple linear model, respectively, after inspection of the general trend between explanatory and predictor variables. Homogeneity of variances was graphically explored by means of residuals versus fits and normal Q‐Q plots. Species abundance data were log‐transformed to meet the assumptions of normality and homoscedasticity.

For the diurnal survey, we explored the general thermal patterns averaging maximum rock temperatures recorded at different seasons in both habitats. Thus, we tested between‐habitat differences in maximum substrate temperature recorded at different times or phases (hours), and averaged across seasons (i.e., four daytime samplings conducted at four different seasons), using a mixed effect model with habitat and daytime as fixed factor and the sampling replicates as random factor. Thus, we considered a random intercept model obtained with a maximum likelihood estimate (Zuur et al., 2009). Pseudo‐*R* ^2^ as coefficients of determination was estimated as before. Analyses were made using the library *MASS*, *lme4* and *lmerTest* in the R‐environment (R Core Team, 2018).

To examine the variation in substrate temperature mapped in the different positions of boulders and rip‐raps in the localities of Coquimbo (29°S) and Iquique (20°S), we estimated the coefficient of variation (CV) for each position and habitat pooling for all sampling dates. We estimated the confidence intervals (95%) of the coefficient of variation through bootstrapping our results and assuming a normal distribution of our data sets (Manly 1998).

## 3 RESULTS

### 3.1 Spatial–temporal thermal patterns and environmental variables

**Figure 2:**
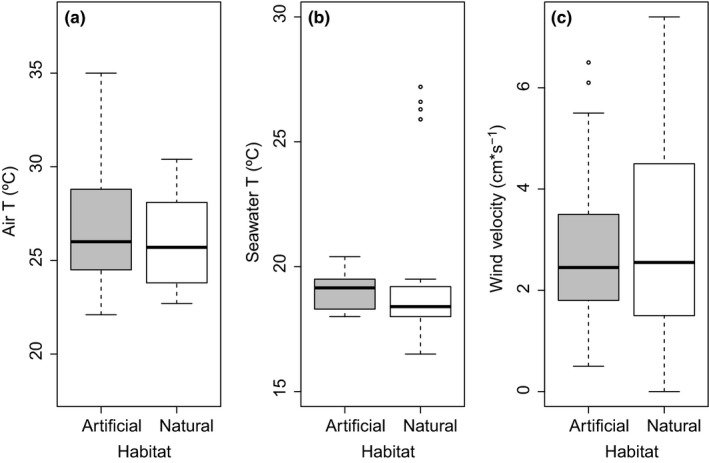
Box plot of (a) the substrate (ST), (b) air (Air T) and (c) seawater temperature, and (d) wind velocity, estimated in rip‐raps (Artificial) and boulder field (Natural) at the different sites considered. The black line in each box is the median, the boxes define the hinge (25, 75% quartile, and the line is 1.5 times the hinge). Points outside the interval (outliers) are represented as dots

Most topographic and environmental variables were relatively similar between the habitats types considered with the exception of the structural complexity (large scale), which was variable among sites and different between habitat types (Figure S2A,B, Table S1 in Appendix). Artificial rip‐raps had a relatively higher complexity than natural boulder fields, but this was dependent on locality (i.e., two localities were different while the other not; see Figure S2B). Similarly, small‐scale topographic complexity was similar between habitat types (Figure S2D). There were significant negative effects of topographic complexity (small scale) and slope on average minimum substrate temperature in both rip‐raps and boulders (Table S2). Air temperature was higher in the artificial than the natural habitat (Figure 2a), which was variable across sites (Table S3a). Substrata temperature had a significant effect on air temperature, but explained very little of the variability observed across habitats and sites (*R* ^2^ = .0597; *n* = 321, *SE* = 2.96). No differences in seawater temperature nor wind velocity were observed between habitat types (Figure 2b and c, Table S3c in Appendix).

**Figure 3:**
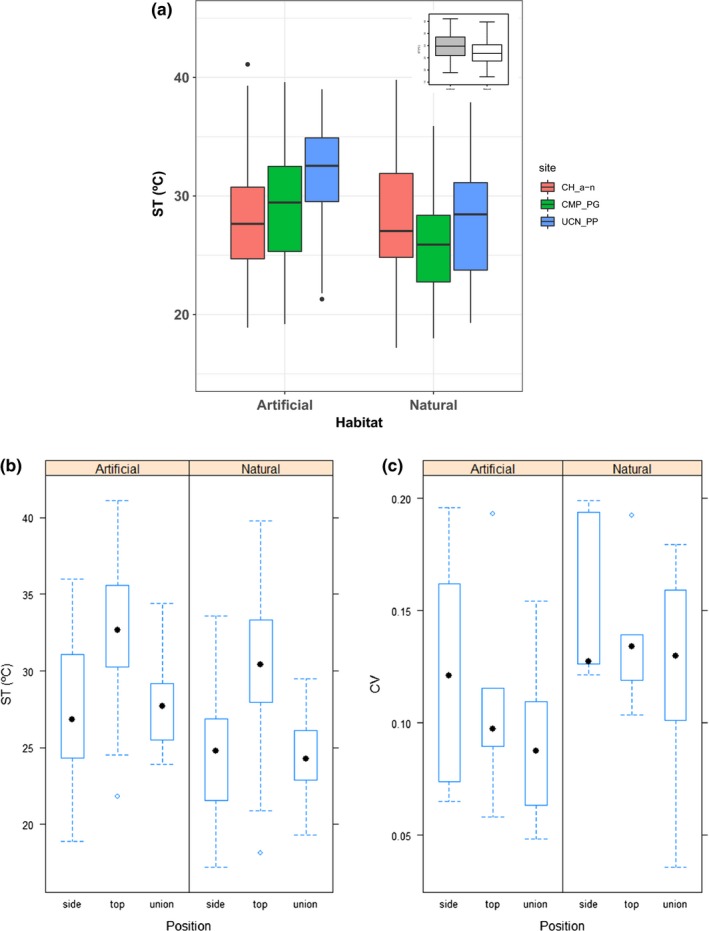
Box plot of (a) the rock or substrate temperature (ST) recorded at the different habitats across localities, and (b) substrate temperature and (c) coefficient of variation (CV) recorded at the different positions on the rip‐raps and natural boulders. Insert in A shows a box plot with the total average rock temperature recorded for the artificial and natural habitat in all localities considered in Coquimbo (30°S). The black line in each box is the median, the boxes define the hinge (25% and 75% quartile, and the line is 1.5 times the hinge). Points outside the interval (outliers) are represented as dots. Sites in (a) correspond to artificial breakwaters (CH_a, CMP, UCN) and corresponding natural boulder fields (CH_n, PG, PP) considered in the “artificial–natural” habitat comparisons

Substrate temperature was variable among localities and was, on average, about 1.5–5°C higher in the artificial habitats (Figure 3a). Median substrate temperature was 33°C with a maximum of 35°C in the rip‐raps, while in natural boulders, median values fluctuated between 25–27°C (see insert in Figure 3a). Substrate temperatures varied significantly within the different positions sampled on rip‐raps and boulders (Figure 3b), with the top of rip‐rap having higher substrate temperatures (ST) than “side” and “union” positions (Figure 3b). The “side” and “union” positions had similar averaged substrate temperature (ST_mean), which were significantly lower in the natural than the artificial habitat (Figure 3b, Table S4), especially in the CMP‐PG comparison, where the ST_mean was significantly lower at compared with the others artificial‐natural sites pairs (Figure 3a, Table S4). In contrast, no differences were observed between habitat at Caleta Hornos (CH_n‐a_, Figure 3a). Habitat and position (fixed effects) contributed thus about 50% of the variation of substrate temperature (marginal pseudo‐*R* ^2^ = .468). In general, the ST_mean recorded across different zones of boulders and at the different localities was less variable in the artificial habitat for all positions considered as indicated by the lower median coefficient of variation estimated (black dot in Figure 3c and see Table S5).

**Figure 4:**
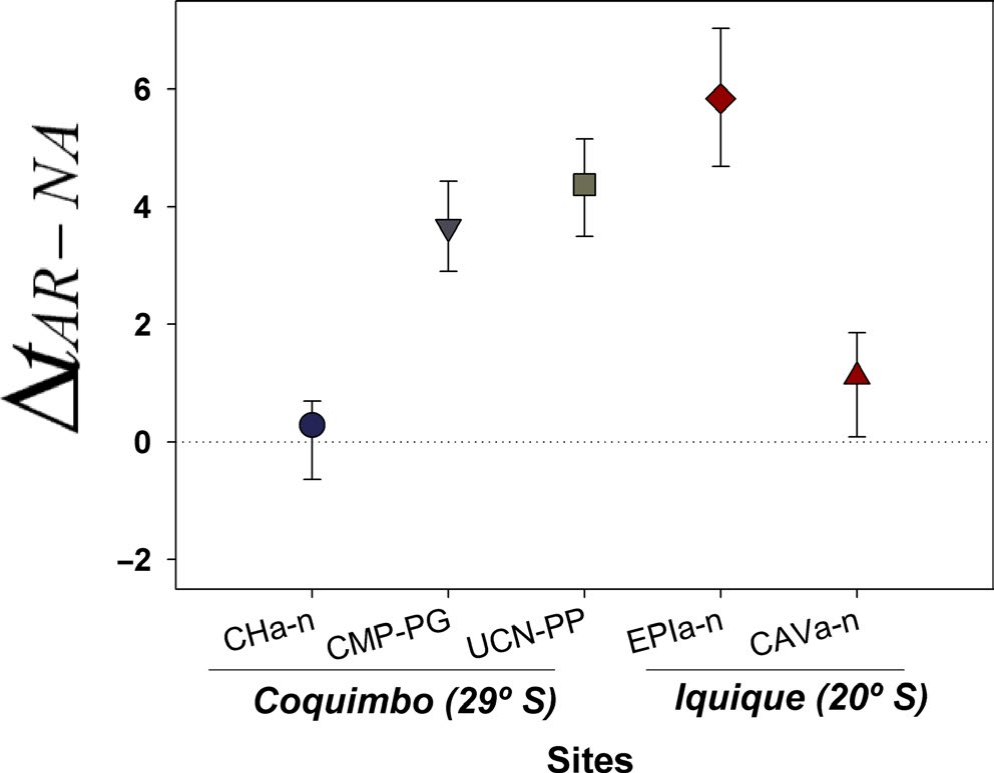
Differences in substrate temperature (°C) between the artificial (AR) and the natural habitat (NA) for the different sites considered (CH_n_, PG, PP, EPI_n_, and CAV_n_ correspond to the natural habitats, CH_a_, CMP, UCN, EPI_a_ and CAV_a_ are the artificial breakwaters considered at the different localities). Bars are 95% confidence intervals estimated through a bootstrapping procedure. Positive values show net increase in substrate temperature in the artificial habitat compared with the natural one. Red diamond and triangle are sites located in Iquique, at northern Chile (20°S)

Net differences in substrate temperatures between the artificial and the natural habitat recorded within each quadrat at the different natural habitat/rip‐rap position considered, showed contrasting patterns. At Coquimbo (29°), at two sites, the artificial habitat showed a net and significant (i.e., 95% CI do not cross zero) increase in substrate temperature compared with the natural (adjacent) habitat (grey symbols in Figure 4) with, on average, substrate temperature being about 3.9–5.0°C higher than the natural habitat. At Caleta Hornos (CH_a‐n_), however, there was no recorded difference in substrate temperature (Figure 4). At the two additional localities sampled in Iquique (19°S), the artificial habitat had significantly higher rock temperature compared with the natural habitat (red symbols in Figure 4). Even at EPI_a‐n_ site in Iquique, we found the largest average difference in rock temperature (i.e., 5.9°C, Figure 4).

**Figure 5:**
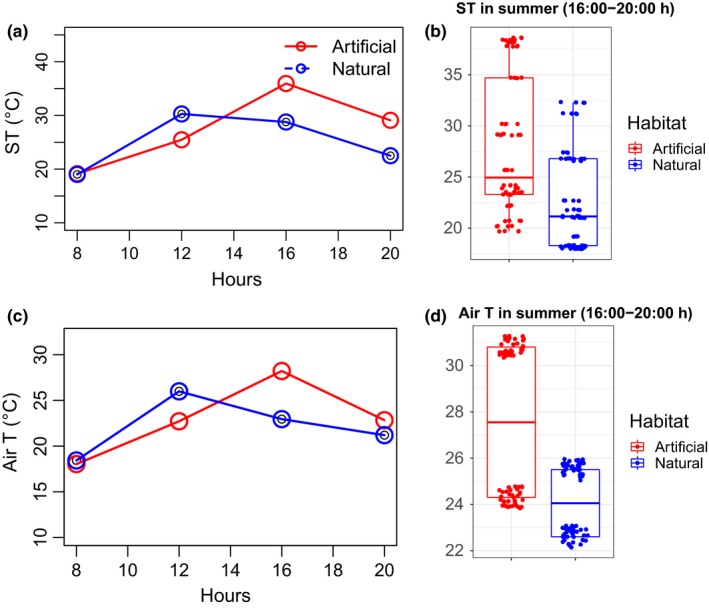
Substrate and air temperatures recorded during daytime sampling (*n* = 37 estimations per hour per habitat in each season). (a) Maximum averaged (4‐seasons) substrate temperature recorded at different hours in both the artificial and the natural habitats, (b) boxplot of the maximum substrate temperature recorded at afternoon and evening (16:00–20:00 hr) in summer, (c) average (4‐seasons) air temperature recorded at different hours in both the artificial and the natural habitats (0.5–1.5 m above the substrata), and (d) boxplot of the air temperature recorded at afternoon and evening (16:00–20:00 hr) in summer. The line in each box is the median, the boxes define the hinge (25% and 75% quartile, and the line is 1.5 times the hinge). Points outside the interval (outliers) are represented as dots

Maximum averaged (over the four seasons) daytime substrata temperature (ST_max) recorded at La Herradura in Coquimbo were variable in both habitats, peaking at mid‐day (12:00 hr; 30°C) for the boulder fields and at 16:00 hr for the artificial habitat (~37°C, Figure 5a). Rock temperature was about 30°C at 20.00 hr at evening in the artificial habitat (red line in Figure 5a). This pattern was more evident during summer, when median substrate temperature recorded during the afternoon and evening (i.e., 16:00 and 20:00 hr pooled data; Figure 5b) was about 5°C higher in the artificial than the natural habitat (Figure 5b). Despite temperature differences being less marked during early morning and mid‐day, a significant effect of habitat (natural) × time was observed (see Table S6 in Appendix). The “Conditional” pseudo‐*R* ^2^ (0.201) suggest an important effect of both fixed but also random effects (plots) altogether instead of considering the fixed effects alone in the model (“Marginal” pseudo‐*R* ^2^ = 0.172). The overall daily pattern in substrate temperature was consistent between summer and winter, but was less evident during autumn in which differences were less marked (see Figure S5 in Appendix). Average daytime variation in air temperature (0.5–1.5 m above the substrata) showed a pattern consistent with that of the substrate temperatures, thus showing higher air temperatures in the artificial habitat than the natural one (Figure 5c,d). Differences in air temperatures between the artificial and the natural habitat were, however, more marked in summer during the afternoon and evening (16:00–20:00 hr), compared with the artificial habitat being about 3.5°C higher (median values) than the natural habitat (see Figure 5d).

### 3.2 Species abundance and richness

**Figure 6:**
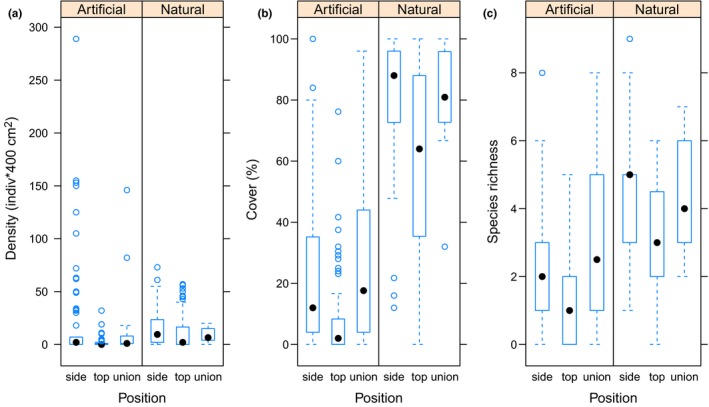
Biotic variables recorded in natural and artificial habitats. (a) Box plot of the density of mobile species (*n* = 64 quadrats per habitat), (b) percent cover of sessile invertebrates and algae, and (c) total species richness (number of species) recorded at the different quadrats located in different positions within the natural and the artificial habitats

Species abundance and richness were variable in the different positions and habitats considered (Figure 6). Mobile species densities were reduced in the artificial habitat, although littorinid snails such as *Echinolittorina peruviana* and *Austrolittorina araucana* reached large densities accounting for most of the mobile species density recorded in this habitat (see outliers in “side” in Figure 6a). There was a weak, nonlinear negative relationship between mobile species density with substrata temperature (Polynomial order 2; *R* ^2^ = .049; *SE* = 27.19; *p* = .0001). Both algae and sessile invertebrate species cover (%) was lower in the artificial habitat as compared with the natural habitat but depending on specific comparison (Table S7a in Appendix). Thus, cover on “top” of rip‐raps had significantly lower cover than “sides” or “unions” (Figure 6b). In general, bare rock cover reached about 85% in the artificial habitat. Barnacles (*Jehlius cirratus*), mussels (*Perumytilus purpuratus*) and noncalcareous algae like *Hildenbrandia lecanelleri* were, however, abundant in the natural habitat reaching about 80%–90% on the “sides” and “unions” of boulders. In contrast, no mussels were observed in the rip‐raps and cover of barnacles and algae was low (less than 30% in average). It should be noted that, despite the general trends was that the artificial habitats had lower species abundances, some natural habitats (e.g., in PG natural site) had also reduced species cover (see negative coefficients in Table S7a). Species richness was lower in the artificial versus the natural habitat for the different within boulder or rip‐raps position considered (Figure 6c). Average substrate temperature (ST_mean) had a significant negative effect on species richness recorded in the study (Table S8).

## 4 DISCUSSION

Field sampling showed strong spatial variation in the thermal patterns in artificial as well as natural habitats, with the top position of boulders having higher temperatures than either sides or between‐boulder unions. Small‐scale spatial complexity (few cm) was similar between habitat types and was associated with lower substrate temperature, suggesting an important role of topographic elements (e.g., between‐boulders, rip‐raps interstices) in reducing heat for intertidal organisms. In general, the artificial habitat experienced higher average and maximum substrate temperatures than the natural habitats, but showed less spatial variability, being about 3.7°C higher in four out of the five study sites in Coquimbo (29°S) and Iquique (20°S). Importantly, both sessile and mobile species abundance and richness were negatively related with peaks of substrate temperature. Specifically, thermal pattern found in artificial infrastructures like rip‐raps in the mid‐to high intertidal level were associated with reduced abundance and species richness. Between‐habitat differences in thermal patterns were persistent during the day and maintain relatively similar at different seasons, with the substrate temperature recorded in the artificial infrastructure being higher in the afternoon. Our study is among the first to report the thermal patterns of coastal artificial infrastructures and their potential consequences on biodiversity patterns. While more information is required to determine how substrate thermal patterns influence microclimatic conditions on urbanized coasts, it seems that artificial infrastructures like rip‐raps may contribute to local coastal warming.

### 4.1 Higher substrate temperature in artificial habitats and consequences for biodiversity

Reduced topographic heterogeneity in artificial infrastructures is assumed to account for the reduction and variation in species abundance and diversity (Firth et al., 2013; Loke et al., 2015). Differences in spatial complexity at small scales (few cm) between artificial and natural habitats are, therefore, well correlated with species occurrences due to the presence or absence of refuges to reduce desiccation stress like shaded crevices, or rock pools (Aguilera et al., 2014; Firth et al., 2013). In our study, we found relatively higher species abundance and species richness in the natural versus the artificial habitats, a pattern commonly observed in different coasts worldwide (e.g., Firth et al., 2016; Firth et al., 2014; Perkins et al., 2015). We also observed a significant negative effect of substrate temperature on sessile species cover and species richness, with both variables showing a pattern consistent with the between‐boulder spatial variation in substrate temperature. In addition, we observed lower variation in substrate temperature along the artificial infrastructure. Interestingly, despite small‐scale (few cm) substrate complexity showed a negative relationship with rock temperature, this was similar in both habitats. However, large‐scale complexity (at the scale meters) was higher in the artificial habitat suggesting that cavities or interstices among rip‐raps were larger than in the natural boulder fields. It seems improbable, however, that small‐ or large‐scale complexity can account for the observed differences in rock temperature. In this context, substrate roughness, and/or boulder shape may have a role limiting/allowing substrate heat absorbance/reflectance (recorded by the thermal camera) and/or propagule settlement contributing to thermal variability and, as a consequence, species occurrences. Substrate roughness may also vary with age, according to decomposition of material through time especially in artificial structures. In our study localities, rip‐raps were built in relatively similar years (Coquimbo: 2003–2004; Iquique: 2005), and some degree of erosion could be expected on them. Substrate roughness can promote effective settlement of algae and sessile invertebrates (Coombes et al., 2015) by increasing moisture and thus reducing substrate temperature. In addition, rough and textured surfaces reflect light less efficiently, and have a higher emissivity than smooth ones (Seuront et al., 2018). Boulder topography, for instance, could account for differences in exposure to sun heating or shading potential (Chapman, 2012; Liversage, 2016). Artificial boulders or rip‐raps tend to be “block‐shape,” while natural boulders are more “round” (ovoid in shape), and thus will experience different exposure to insolation, heat absorbance/reflectance and then emissivity (Lathlean et al., 2017; Seuront et al., 2018). In our study, rocks present at the rip‐raps and natural boulder field, were characterized by a relatively similar concentration of plagioclase and quartz, biotite, and hornblende mineral. However, geochemical differences may account for differences in thermal absorbance patterns (Lathlean & Seuront, 2015), and could be important in other locations or latitudes and should be considered in further studies. In addition, granite boulders could be more alkaline or acid (lower pH) than natural boulders which could account for differences in temperature and/or biodiversity. We did not measure rock pH in the field and, at known, no studies have provided information about comparison of acidity in rip‐raps versus natural rocks and thus we do not speculate in this context. Given our results, it seems that organisms could experience more thermally stressful conditions (Helmuth et al., 2006; Somero, 2002) in rip‐raps, potentially contributing to the lower occurrences of both sessile and mobile organisms observed in these habitats. It should be noted that absence of foliose algae or mussels, and reduced cover of barnacles in the mid‐high intertidal zone in the artificial habitat may, themselves also contribute to increased thermal stress (Helmuth et al., 2006; Lathlean et al., 2012) as biogenic heterogeneity has an important role providing thermal refuges (Cartwright & Williams, 2012). However, given we did not explore thermal stress of organisms, which requires a complementary and/or more appropriated temperature‐recording methodology (Judge et al., 2018), we can only speculate in this context. Notwithstanding, it should be noted that presence/absence of sessile species may also reduce (e.g., microalgae, foliose algal forms) or even increase (e.g., dark crustose algae) the rock temperature recorded, and thus, a negative or positive temperature‐species cover relationship could be expected.

### 4.2 **Could artificial infrastructures modify coastal microclimate**?

Commonly, in coastal cities, heat extremes are expected to be less severe than in valleys or inland suburbs as sea breezes can regulate heat waves by reducing city temperatures (Kuttler, 2008). Nevertheless, as in other cities, coastal artificial infrastructures have the potential to store‐emit heat (i.e., gain or lose heat) and to contribute to rising air and substrate temperature during sunny days which has been poorly explored previously. In our study, rock temperature was about 3.7–5.8°C higher in the artificial infrastructure than the adjacent natural habitat. In addition, in the artificial infrastructure, the air temperature recorded at 1.0–1.5 m above the substrata was higher than in the natural habitat despite similar wind speeds. It appears, therefore, that rip‐raps effectively maintains higher air temperatures than other neighboring areas, a pattern commonly observed within the urban environment (Larsen, 2015; Oke, 1988). In this context, in our study we observed that air temperature decreased at late evening (during sunset) in the natural boulder field, but maintained >1.5°C higher in the artificial habitat. This pattern was observed in all seasons, but was more evident during summer when air temperatures were about 3.5–4.0°C higher in the artificial habitat. Given that artificial infrastructures made of granite boulders or rip‐raps are frequently associated with other coastal infrastructures such as seawalls, wharfs, roads, and pedestrian walkways, the net effect of these integrated structures on microclimatic conditions at night (by heat release) may contribute to local coastal warming in relatively similar ways as built infrastructures generates urban “heat island effect” in inland cities (Kuttler, 2008; Oke, 1989), a phenomenon that requires further examination in coastal marine habitats.

### 4.3 Ecological engineering solutions for rock temperature increase at artificial habitats

Thermal regulation by green infrastructure is an important concern in urban areas and a matter of critical consideration for urban planning (Gunawardena, Wells, & Kershaw, 2017). Equivalent strategies are recently being implemented in urbanized seascapes (Dafforn et al., 2015; Strain et al., 2018), where an increase of spatial complexity‐heterogeneity (Loke & Todd, 2016) by the addition of rock pools (Browne & Chapman, 2011; Chapman & Blockley, 2009; Evans et al., 2016; Firth et al., 2014; Waltham & Sheaves, 2018), crevices and pits (Martins, Thompson, Neto, Hawkins, & Jenkins, 2010), among other topographic features, to artificial infrastructures has the potential to reduce thermal stress to organisms and are important strategies for rehabilitation of coastal urban systems (Strain et al., 2018). We demonstrate that artificial structures like rip‐raps or granite boulder breakwaters have higher rock temperatures than natural substrates, which can even influence the local air temperature and can contribute to alter abundance and biodiversity patterns. Artificial breakwaters are also thermally more homogeneous at the scale of meters than natural habitats, indicating that intertidal organisms experience a more similar temperature pattern across the artificial habitat with reduced frequency of thermal refuges as in the natural habitats. This pattern could also explain the potential for artificial tilt boxes to provide microclimate ledges in shading zones along artificial breakwater in estuarine systems (Waltham & Sheaves, 2018). Initial practical indications would be executed based on our main results, to reduce or mitigate enhanced rock temperature in artificial breakwaters at mid‐high intertidal levels. Choice of material with reduced potential to absorb/accumulate heat would be important during the design stage for construction of artificial structures. In addition, creation of small‐scale (cm) spatial heterogeneity in “top” or “sides” of rip‐raps with construction of grooves, pits, and/or small rock‐pools and transplant of mussels could complementarily increase thermal refuges availability for species settlement and or aggregations (Evans et al., 2016; Firth et al., 2013; Martins et al., 2010; Morris, Golding, Dafforn, & Coleman, 2018; Strain et al., 2018). In addition, “among rip‐raps” union, spaces or interstices would be modified through installing rock pools (Browne & Chapman, 2011; Waltham & Sheaves, 2018) to enhance water retaining features in a proportion of the breakwaters (e.g., ~10%) high enough to reduce overall structure heat gain. Ecological engineering solutions to enhance biodiversity and values for ecosystem functioning in artificial infrastructures, should consider the complex spatial‐temporal thermal structure of these novel habitats in their research and planning agendas. Therefore, future studies could consider complementary methodologies to deal with variation of thermal patterns at different spatial scales (e.g., see Judge et al., 2018; Lathlean & Seuront, 2015 for reviews), in a suite of integrated coastal artificial infrastructures. This could shed light on the potential large‐scale effect of coastal urban infrastructures in contributing to exacerbate the local effect on biota of frequent heat waves, and in the subsequent alteration of the coastal climate.

## CONFLICT OF INTEREST

The authors declare no competing interests.

## AUTHOR CONTRIBUTIONS

MAA conceived the ideas and designed the surveys, analyzed the data, and led the writing of the manuscript. RMA and TM conducted the surveys and revised the manuscript.

## Data Availability

Data are accessible in Dryad data repository: https://doi.org/10.5061/dryad.44j0zpc8v
