# Supplementary material for: Mapping microhabitat thermal patterns in artificial breakwaters: Alteration of intertidal biodiversity by higher rock temperature
Source: Ecol Evol. 2019 Nov 4;9(22):12915–27. doi: 10.1002/ece3.5776 (PMC6875675; doi:10.1002/ece3.5776)
Supplement: Supplementary file 1 [file ECE3-9-12915-s001.docx]

**Supporting Information**

**Appendix of Figures &Tables**

**Mapping microhabitat thermal patterns in artificial breakwaters: alteration of intertidal biodiversity by higher rock temperature**

Moisés A. Aguilera*, René M. Arias, Tatiana Manzur

*Departamento de Biología Marina, Facultad de Ciencias del Mar, Universidad Católica del Norte, Larrondo, 1281, Coquimbo, Chile*

**Figure S1.** General view of natural boulder field and an artificial breakwater (rip-rap) considered in the study, and details of substrate characteristics of a natural boulder and a rip-rap. The red frame area is 40 cm^2^.


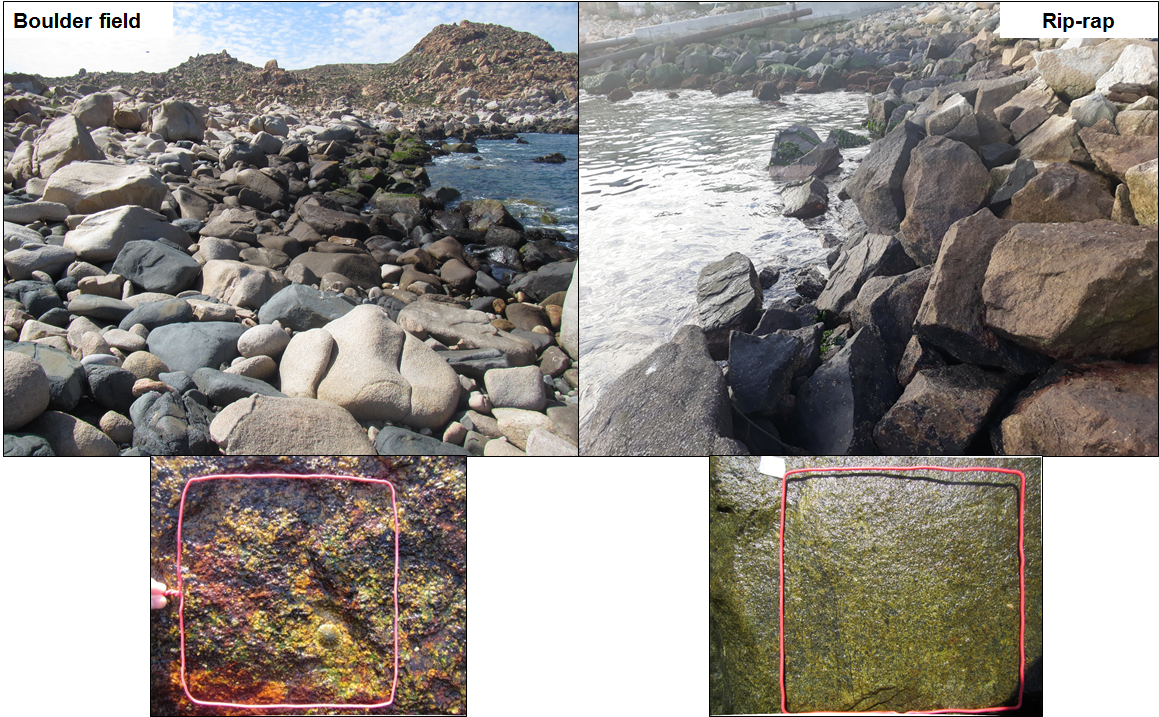


**Figure S2.** Scheme of natural boulders-rip-rap sampling protocol (A, C), and structural complexity measured across sites B) and inside quadrats D) in artificial (rip-raps) and natural (boulder field) habitats. In A, blue lines correspond to chain (fitted to substrate) measures while read line corresponds to linear measures (1m). The ratio between the chains to linear measures was used as an estimation of structural complexity in each habitat. In B, “side” and “top” correspond to specific locations within individual rip-raps and natural boulders, while “union” correspond to interstices generated by two or more rip-raps or boulders close together.


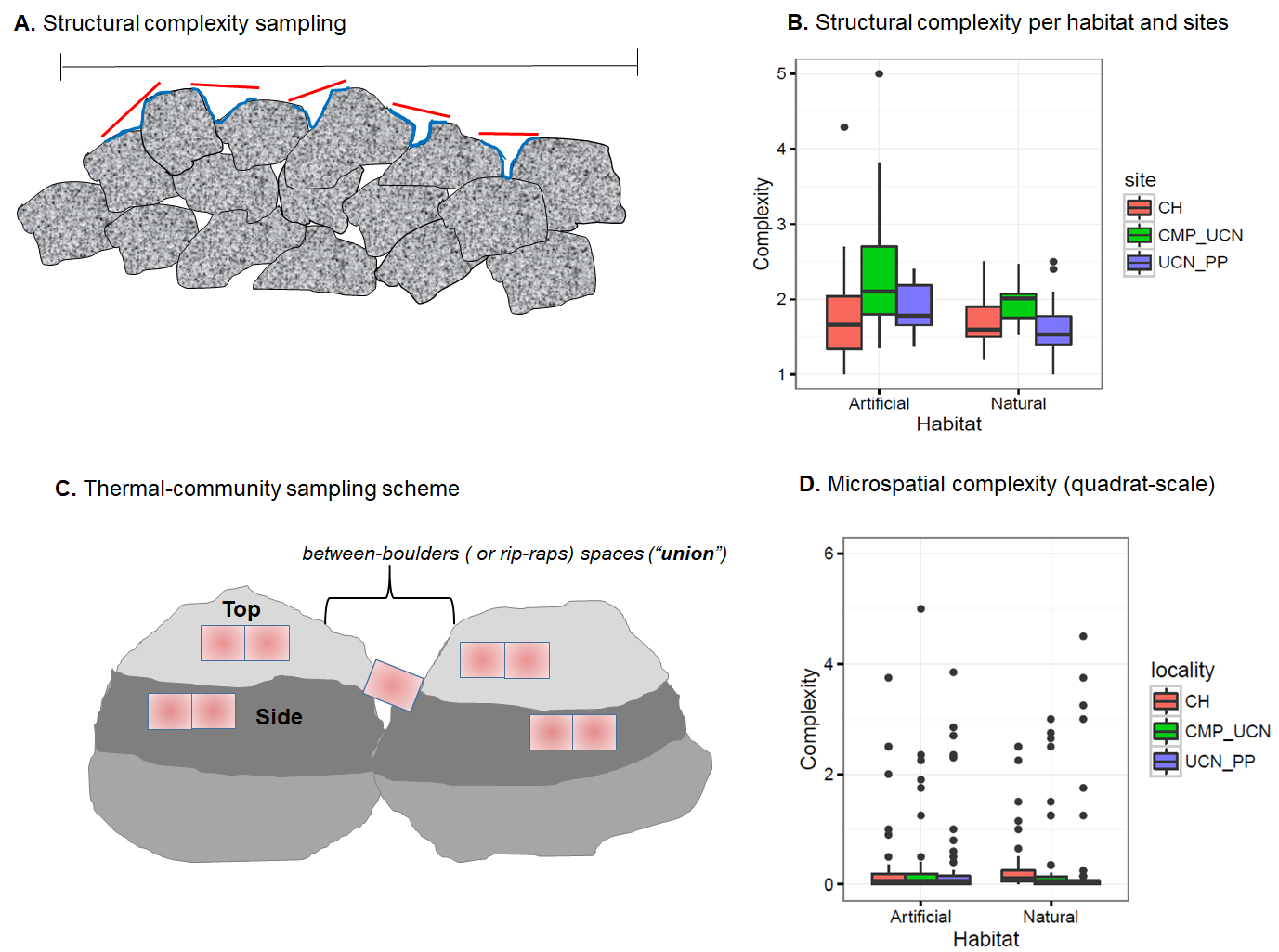


**Figure S3.** Polar plot of wind velocity (m*sec^-1^) and direction estimated in the different habitats and localities during the thermal sampling


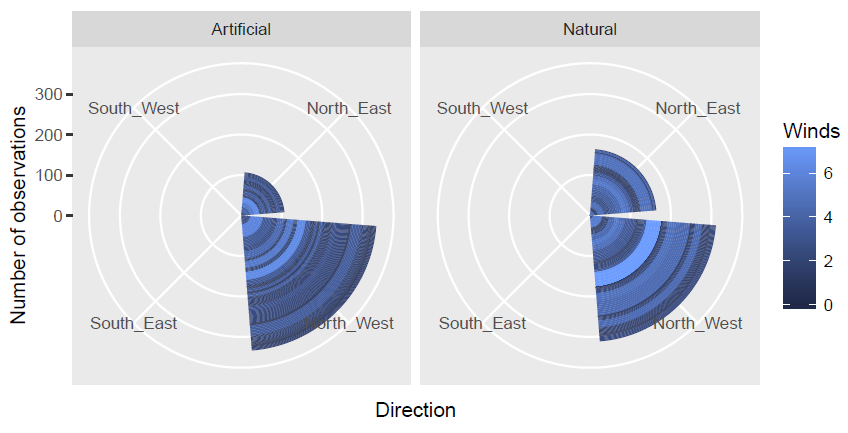


**Figure S4.** (A) Seascape and (B) small-scale (individual Rip-rap) thermal pictures of artificial breakwaters (e.g. at UCN site), and representation of thermal pixels-data extraction for analyses from individual thermal snapshot (C and D. The black frame in C and D show the quadrat utilized for sampling (20 x 20 cm).


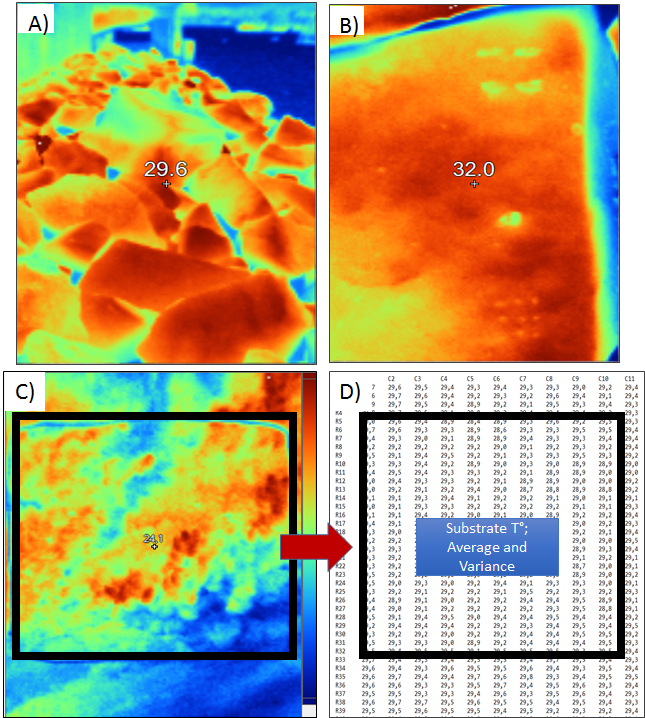


**Figure S5.** Daytime sampling of substrate temperature (°C) conducted in UCN and PG sites at La Herradura Bay from summer to spring 2018.


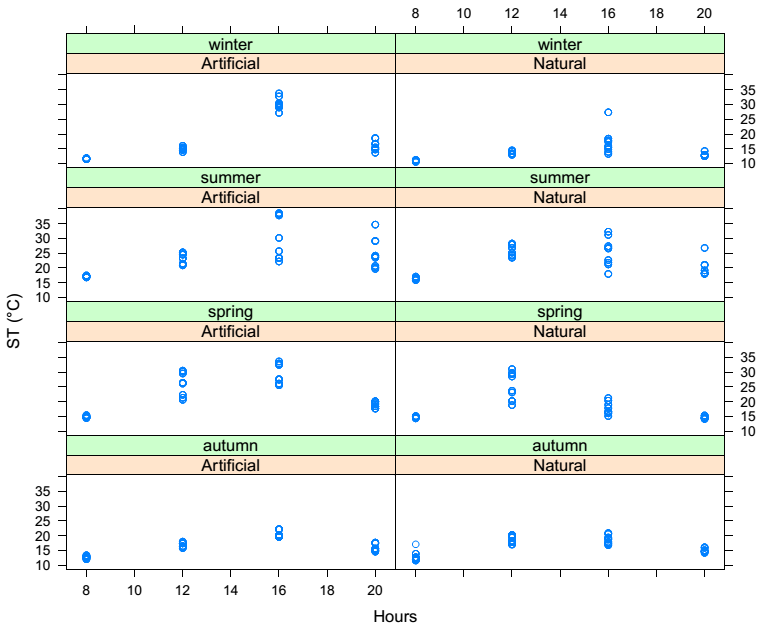


**Tables**

**Table S1.** Two-way nested ANOVA on structural complexity estimated between natural and artificial habitat at the different study site.

| **SV** | **df** | **MS** | **F** | **P** |
| --- | --- | --- | --- | --- |
| Habitat =H | 1 | 10.53 | 39.81 | **<0.0001** |
| Site=S(H) | 1 | 0.074 | 0.281 | 0.598 |
| Residual | 102 | 0.265 |  |  |

**Table S2.** Summary of a linear model on effects of rock slope and small-scale complexity (few cm) on minimum substrate temperature (ST_min), recorded at different position on natural and artificial habitats at different study sites.

|  | **Estimates** | **Std. Error** | **t-value** | **P** |
| --- | --- | --- | --- | --- |
| *Intercept* | 21.31 | 0.303 | 70.33 | **<0.0001** |
| Complexity=C  Slope=S | -0.721  -0.0155 | 0.215  0.0072 | -3.357  -2.134 | **0.0009**  **0.0336** |
| C*S | -0.0159 | 0.0116 | -1.370 | 0.1716 |
| *Linear model statistics* | **R^2^** | **Residual Std. Error** | **df** | **P** |
|  | *0.10* | 2.621 | *320* | ***<0.0001*** |
|  |  |  |  |  |

**Table S3.** Summary of ANOVA on **a)** air and b**)** Seawater temperature (°C), and **c)** wind intensity (m*sec^-1^), recorded in both natural and artificial habitats at different study sites. Factor “site” was considered random and nested on habitat type. Data for seawater temperature were log+1 transformed to improve variance homogeneity.

|  |  | |  |  |  |
| --- | --- | --- | --- | --- | --- |
| **SV** | **Df** | | **MS** | **F** | **P** |
| **a) Air** |  | |  |  |  |
| Habitat=H | 1 | | 41.89 | 6.189 | **0.0134** |
| Site (H) | 4 | | 197.48 | 29.177 | **<0.0001** |
| Residual | 318 | | 6.77 |  |  |
|  |  | |  |  |  |
|  |  | |  |  |  |
| **b) Seawater** |  | |  |  |  |
| Habitat=H | 1 | | 0.018 | 3.387 | 0.0666 |
| Site (H) | 4 | | 0.449 | 84.4 | **<0.0001** |
| Residual | 318 | | 0.0053 |  |  |
|  |  | |  |  |  |
|  | |  |  |  |  |
| **c) Wind velocity** | |  |  |  |  |
| Habitat=H | 1 | | 4.69 | 2.134 | 0.145 |
| Site (H) | 4 | | 37.66 | 17.11 | **<0.001** |
| Residual | 318 | | 2.20 |  |  |
|  |  | |  |  |  |

**Table S4.** Summary of linear mixed effect model on average rock temperature (ST_mean) recorded at different positions on natural and artificial habitats at different sites study sites at Coquimbo; CMP, UCN, CH_a_ and PG, PP, CH_n_ denote specific location of artificial breakwaters and corresponding natural habitat, respectively. Plots correspond to boulders and rip-raps groups sampled in each site at each habitat type. Significant P-values are presented in bold.

|  | Coeff | StdError | t-value | P |
| --- | --- | --- | --- | --- |
| *Fixed effects* |  |  |  |  |
| Intercept | 28.9750 | 0.8213 | 35.281 | **<0.001** |
| Habitat (Natural; NA) | 1.4167 | 1.1599 | 1.221 | 0.222 |
| Position (Top; Pt) | 6.1500 | 1.1615 | 5.295 | **<0.001** |
| Position (Union; Pu) | 4.5250 | 1.1615 | 2.465 | **0.001** |
| NA×Pt | 0.7708 | 1.8354 | 0.470 | 0.639 |
| NA×Pu | -4.4333 | 1.6403 | -1.709 | 0.088 |
| AR×CMP | 1.2333 | 2.5935 | 1.051 | 0.297 |
| NA×PG | -3.1583 | 1.1732 | -2.692 | **<0.001** |
| AR×UCN | 5.7167 | 1.1732 | 4.603 | **<0.001** |
| NA×PP | -2.3458 | 1.2419 | -1.889 | 0.084 |
| AR×Pt×CMP | 0.9167 | 1.2419 | 0.552 | 0.582 |
| NA×Pt×UCN | -3.1792 | 1.6592 | -1.916 | 0.059 |
| AR×Pu×CMP | -0.3000 | 2.6115 | -0.115 | 0.909 |
| NA×Pu×PG | 4.4083 | 2.6115 | 1.688 | 0.093 |
| AR×Pt×UCN | -3.4583 | 1.7564 | -1.969 | 0.073 |
| NA×Pt×PP | 0.2042 | 1.7564 | 0.116 | 0.909 |
| AR×Pu×UCN | -4.8667 | 2.7051 | -1.799 | 0.083 |
| NA×Pu×PP | 3.5625 | 2.7051 | 1.317 | 0.199 |
|  |  |  |  |  |
|  | Variance | Std.Deviance |  |  |
| *Random effects* |  |  |  |  |
| Group (Intercept) | 0.0037 | 0.0609 |  |  |
| CMP | 0.0624 | 0.2497 |  |  |
| UCN | 0.3942 | 0.6279 |  |  |
| Residual | 16.1432 | 4.0178 |  |  |
|  |  |  |  |  |

**Table S5.** Coefficient of variation of mean substrate temperature ST_mean recorded for the different position of natural boulders and artificial rip-raps. 95% confidence intervals were estimated through a bootstrap procedure.

| **Habitat** | **Position** | **CV_boot** | **Ci +95%** | **Ci -95%** |
| --- | --- | --- | --- | --- |
| Artificial | Side | *0.123* | *0.0861* | *0.1629* |
| Artificial | Top | *0.1085* | *0.0833* | *0.1499* |
| Artificial | Intersticies | *0.0918* | *0.0687* | *0.12222* |
| Natural | Side | *0.149* | *0.1255* | *0.l835* |
| Natural | Top | *0.1371* | *0.1198* | *0.1622* |
| Natural | Intersticies | *0.1225* | *0.0759* | *0.1562* |
|  |  |  |  |  |

**Table S6.** Summary of linear mixed effect model for maximum substrate temperature (ST_max; °C) recorded at both natural and artificial habitats during the day (‘daytime sampling’) at different season. Significant values (α=0.05) are presented in bold.

|  | Coeff | Std.Error | t-value | P |
| --- | --- | --- | --- | --- |
| *Fixed effects* |  |  |  |  |
| Intercept | 13.5706 | 0.9595 | 14.143 | **<0.001** |
| Habitat (Natural; NA) | 5.5563 | 1.3497 | 4.117 | **<0.001** |
| Time | 0.8123 | 11.2041 | 10.060 | **<0.001** |
| NA×time | -0.6668 | 0.0919 | -7.251 | **<0.001** |
|  |  |  |  |  |
|  | Variance | Std.Deviance |  |  |
| *Random effects* |  |  |  |  |
| Group (Intercept) | 0.0643 | 0.2563 |  |  |
| Residual | 48.4115 | 6.9578 |  |  |
|  |  |  |  |  |
|  | Intra | NA | Time |  |
| *Intra-class Correlation* |  |  |  |  |
| NA | -0.701 |  |  |  |
| Time | -0.830 | 0.540 |  |  |
| NA×Time | 0.667 | -0.953 | -0.566 |  |

**Table S7.** Summary of lineal regression model on species abundance. a) Mobile species density (log+1 transformed) and b) sessile species cover (%), recorded at different position within natural (boulder field) and artificial (rip-raps) habitats at different study sites at Coquimbo; CMP, UCN, CH_a_ and PG, PP, CH_n_ denote specific location of artificial and natural habitat, respectively. Post-hoc comparison were made with Tukey HSD test. Only significant within (positions) and between habitat (natural versus artificial) differences are presented. Significant values (α=0.05) are presented in bold. AR; Artificial, NA: Natural. Positions: Pt; top; Pu; union.

|  | Estimate | Std.Error | t-value | P |
| --- | --- | --- | --- | --- |
| **a) %Cover sessile** |  |  |  |  |
| Intercept | 19.271 | 3.465 | 5.562 | **<0.001** |
| NA | 66.745 | 4.900 | 13.620 | **<0.001** |
| Pt | -14.418 | 3.566 | -4.044 | **<0.001** |
| Pu | 5.609 | 5.638 | 0.995 | 0.321 |
| NA×Pt | -6.130 | 5.042 | -1.216 | 0.225 |
| NA×Pu | -6.169 | 7.973 | -0.774 | 0.439 |
| AR×CMP | -3.837 | 4.117 | -0.932 | 0.352 |
| NA×PG | -17.015 | 4.117 | -4.133 | **<0.001** |
| AR×UCN | 14.575 | 4.117 | 3.540 | **<0.001** |
| NA×PP | 5.797 | 4.117 | 1.408 | 0.160 |
|  |  |  |  |  |
|  |  |  |  |  |
| **b) Density mobile** |  |  |  |  |
| Intercept | 2.6709 | 0.1702 | 15.687 | **<0.001** |
| Pt | -1.1102 | 0.1752 | -6.337 | **<0.001** |
| Pu | -0.0074 | 0.2770 | -0.027 | 0.978 |
| NA | 0.0319 | 0.2407 | 0.133 | 0.894 |
| NA× Pt | 0.4966 | 0.2477 | 2.005 | **0.045** |
| NA× Pu | -0.1625 | 0.3917 | -0.415 | 0.678 |
| AR×CMP | -1.7458 | 0.2023 | -8.630 | **<0.001** |
| NA×PG | 0.0466 | 0.2023 | 0.231 | 0.817 |
| AR×UCN | -1.6663 | 0.2023 | -8.237 | **<0.001** |
| NA×PP | -1.6489 | 0.2023 | -8.151 | **<0.001** |

**Table S8.** Summary of linear model on the effect of mean substrate temperature (ST_mean) on species richness recorded in both natural and artificial habitats. Significant values (α=0.05) are presented in bold.

|  | **Estimates** | **Std. Error** | **t-value** | **P** |
| --- | --- | --- | --- | --- |
| **Intercept** | 8.716 | 0.539 | 16.16 | **<0.0001** |
| **ST_mean** | -0.206 | 0.0186 | -11.03 | **<0.0001** |
|  |  |  |  |  |
| *Linear model statistics* | **R^2^** | **Residual Std. Error** | **df** | **P** |
|  | *0.274* | *1.702* | *322* | ***<0.0001*** |
